# Curcumin Reverses NNMT-Induced 5-Fluorouracil Resistance via Increasing ROS and Cell Cycle Arrest in Colorectal Cancer Cells

**DOI:** 10.3390/biom11091295

**Published:** 2021-08-31

**Authors:** Guoli Li, Sining Fang, Xiao Shao, Yejia Li, Qingchao Tong, Beibei Kong, Lifen Chen, Yanzhong Wang, Jun Yang, Haitao Yu, Xinyou Xie, Jun Zhang

**Affiliations:** 1 Department of Clinical Laboratory, Sir Run Run Shaw Hospital, School of Medicine, Zhejiang University, Hangzhou 310016, China; liguoli@zju.edu.cn (G.L.); fsnsunny@zju.edu.cn (S.F.); shaoxiao2020@hotmail.com (X.S.); lindsayyejia@163.com (Y.L.); 21918604@zju.edu.cn (Q.T.); kongbaby@126.com (B.K.); clfzjtcm@163.com (L.C.); lanzhijunsx@hotmail.com (Y.W.); yj2020881@163.com (J.Y.); htyu2011@163.com (H.Y.); scottxie@zju.edu.cn (X.X.); 2 Key Laboratory of Precision Medicine in Diagnosis and Monitoring Research of Zhejiang Province, Hangzhou 310016, China

**Keywords:** nicotinamide *N*-methyltransferase, chemoresistance, curcumin, cell cycle arrest, colorectal cancer

## Abstract

Nicotinamide *N*-methyltransferase (NNMT) plays multiple roles in improving the aggressiveness of colorectal cancer (CRC) and enhancing resistance to 5-Fluorouracil (5-FU), making it an attractive therapeutic target. Curcumin (Cur) is a promising natural compound, exhibiting multiple antitumor effects and potentiating the effect of 5-FU. The aim of the present study is to explore the effect of Cur on attenuating NNMT-induced resistance to 5-FU in CRC. A panel of CRC cell lines with different NNMT expressions are used to characterize the effect of Cur. Herein, it is observed that Cur can depress the expression of NNMT and p-STAT3 in CRC cells. Furthermore, Cur can induce inhibition of cell proliferation, G2/M phase cell cycle arrest, and reactive oxygen species (ROS) generation, especially in high-NNMT-expression CRC cell lines. Cur can also re-sensitize high-NNMT-expression CRC cells to 5-FU both in vitro and in vivo. In summary, it is proposed that Cur can reverse NNMT-induced cell proliferation and 5-FU resistance through ROS generation and cell cycle arrest. Given that Cur has long been used, we suppose that Cur is a promising anticancer drug candidate with minimal side effects for human CRC therapy and can attenuate NNMT-induced resistance to 5-FU.

## 1. Introduction

Statistically, colorectal cancer (CRC) is the second most common cause of cancer-related death worldwide [1]. Chemotherapy is still a mainstay of CRC treatment, and adjuvant chemotherapy based on 5-fluorouracil (5-FU) can significantly improve the prognosis of CRC [2]. However, 5-FU resistance occurs in certain areas of CRC patients, which leads to poor clinical prognosis [3]. Therefore, the discovery of drugs in possession of a potential synergistic effect with 5-FU is urgently required.

Nicotinamide *N*-methyltransferase (NNMT, E.C. 2.1.1.1) was originally identified as an enzyme that catalyzes nicotinamide to 1-methylnicotinamide (1-MNA) [4]. NNMT has been found highly expressed in a wide variety of cancers, and NNMT over-expression can enhance the aggressiveness of various cancers, including CRC [5]. We previously reported that NNMT could promote cell proliferation, reduce reactive oxygen species (ROS), accelerate cell cycle, inhibit cell apoptosis, and enhance resistance to 5-FU in human CRC cells [6,7]. We also demonstrated that NNMT could promote cell growth, inhibit apoptosis, inhibit autophagy, and enhance chemoresistance in breast cancer [8,9,10]. These data support the notion that NNMT is an attractive tumor marker and therapeutic target. Hence, discovering drugs targeting NNMT and attenuating NNMT-induced 5-FU resistance is critical in improving the prognosis of CRC.

Curcumin (Cur), as a promising natural compound, is a diarylheptanoid belonging to the secondary metabolite from the turmeric plant. Various studies have indicated Cur as a possible therapeutic agent in CRC. The antitumor activity and low toxicity profile of Cur have been demonstrated through multiple cellular pathways involved in regulation of proliferation, apoptosis, survival, angiogenesis, and metastasis [11,12,13,14]. Cur can increase the generation of ROS, which leads to many dysfunctions in cells, including DNA damage, cell apoptosis, and cell cycle arrest in CRC [15,16,17]. Cur also exhibits an antitumor effect as an inhibitor of the transcription factor signal transducer and activator of transcription 3 (STAT3) in various tumors [18,19,20,21], and STAT3 is reported to upregulate the expression of NNMT in CRC [22]. In addition, it has been reported that Cur potentiates the effect of many chemotherapy drugs, including 5-FU [18,23,24]. It has been observed that, regardless of the drug that it is combined with, Cur always results in an enhanced effect with reduced dose and side effects [23].

Given that NNMT can induce 5-FU resistance and Cur can depress NNMT expression, as well as potentiate the effect of 5-FU, this study aims to assess the effect of Cur on NNMT-related 5-FU resistance. In this study, we use a panel of CRC cell lines with different NNMT expressions to characterize the effect of Cur on reducing NNMT-induced 5-FU resistance. Cur treatment generally results in stronger inhibition of cell proliferation and higher levels of arrest at the G2/M checkpoint in high-NNMT-expression CRC cell lines than in low-NNMT-expression CRC cells. We also note that Cur can inhibit NNMT expression and the phosphorylation of STAT3 (p-STAT3) in CRC cells. Moreover, Cur can induce more ROS in high-NNMT-expression CRC cell lines than in low-NNMT-expression CRC cell lines. These data support the notion that Cur can reverse NNMT-induced cell proliferation by increasing ROS and cell cycle arrest. We also observe that Cur can induce cell apoptosis, but the protection of NNMT on cell apoptosis still exists. In addition, we note that Cur potentiates the effect of 5-FU on CRC both in vitro and in vivo.

In summary, we propose that Cur is a promising anticancer drug candidate with low side effects for human CRC therapy and can attenuate NNMT-related resistance to 5-FU.

## 2. Materials and Methods

### 2.1. Cells and Cell Culture

Human CRC cell lines SW480, which exhibited low NNMT expression, and HT-29, which exhibited low NNMT expression, were purchased from Cell Bank at the Chinese Academy of Sciences (Shanghai, China). STR authentication of all cell lines was completed. The modified cell lines SW480/NC and SW480/NNMT (NNMT overexpression) were constructed as described in our previous study [6]. All of the cell lines were cultured in RMPI-1640 (cat. No. 11875-093. Gibco, Grand Island, NY, USA) supplemented with 1% penicillin–streptomycin liquid (cat. No. 15140-122. Merck KgaA) and 10% fetal bovine serum (cat. No. 26010066. Thermo Fisher Scientific, Inc., Waltham, MA, USA) at 37 °C with 5% CO_2_.

### 2.2. Chemical Reagents and Antibodies

Cur (cat. No. S1848, 99.83% purity, Selleck Chemical, Houston, TX, USA) and 5-FU (cat. No. V900394, reagent grade, ≥99%, Merck KgaA) were dissolved in DMSO (cat. No. D8418, Merck KgaA) and stored at −80 °C. *N*-acetyl-l-cysteine (NAC; cat. No. ST1546, Beyotime Biotechnology, Shanghai, China) was dissolved in deionized water. Cur and 5-FU were diluted in culture medium for each experiment, and the final concentration of DMSO did not exceed 0.3%. The mouse anti-human NNMT monoclonal antibody was prepared as previously described [8]. The anti-β-actin (cat. No. 4970), anti-phospho-Stat3 (Tyr705) (cat. No. 9145), anti-p21 (cat. No. 2947), anti-CDK2 (cat. No. 2546), anti-cyclin A2 (cat. No. 4656), anti-CDK1 (cat. No. 9116), anti-cyclin B1 (cat. No. 4138), anti-phospho-Rb (Ser807/811) (cat. No. 8516), anti-cyclin D1 (cat. No. 2978), anti-CDK4 (cat. No. 12790), goat anti-rabbit IgG (cat. No. 7074), and goat anti-mouse IgG (cat. No. 7076) antibodies were all obtained from Cell Signaling Technology (CST, Danvers, MA, USA).

### 2.3. NNMT Knocked down by Short Hairpin (sh)RNA and Small Interfering (si)RNA

The lentivirus expressed NNMT shRNA was designed and synthesized by Shanghai Genechem Co., Ltd. (Shanghai, China) and transfected to HT-29 cells as previously described [6]. The NNMT siRNA and control siRNA were designed and synthesized by Guangzhou RiboBio Co., Ltd. (Guangzhou, China) and transfected to HT-29 cells as previously described [24].

### 2.4. Cell Viability Assay

Cell viability assay was detected using a Cell-Counting Kit-8 (CCK-8; cat. No. CK04; Dojindo Molecular Technologies, Inc., Kumamoto, Japan) following the manufacturer’s instructions, Briefly, 5 × 10^3^ cells were seeded in 96-well plates per well and then incubated with different concentrations of Cur and/or 5-FU for 24 h. CCK-8 solution (1:10 *v*/*v*) was added to cells and incubated at 37 °C for 2 h, and absorbance at 450 nm was measured using a microplate reader (Bio-Rad, Hercules, CA, USA). The inhibition rate (IR) = [1 − (mean absorbance of experimental wells/mean absorbance of control wells)] × 100%.

### 2.5. Western Blot Analysis

Cells were lysed in RIPA lysis buffer (Beyotime Biotechnology, Shanghai, China) with protease inhibitors to extract total protein. Then, a 40 μg protein sample was separated by SDS-PAGE and transferred to a PVDF membrane (cat. No. ISEQ00010; Millipore, Bedford, MA, USA), followed by immunoblotting with primary antibodies (1:1000 *v*/*v*) and horseradish peroxidase-conjugated secondary antibodies (1:5000 *v*/*v*). Signals were visualized and captured by Image Lab (Bio-Rad, Hercules, CA, USA).

### 2.6. Cell Apoptosis Analysis

The Annexin V-PE/7-AAD Apoptosis Detection kit (cat. no. 559763, BD Biosciences) and Annexin V-FITC/PI Apoptosis Detection kit (cat. no. 556547, BD Biosciences) were used to detect apoptosis according to the manufacturer’s instructions, and signals were analyzed by flow cytometry (FACSCalibur flow cytometer; BD Biosciences, San Jose, CA, USA), as previously described [24].

### 2.7. Cell Cycle Analysis

Cell cycle was analyzed using a cell cycle staining kit (cat. no. CCS01, Multisciences (lianke) Biotech Co., Ltd., Hangzhou, China) according to the manufacturer’s instructions. Briefly, cells were harvested and washed with cold PBS twice, stained with binding buffer containing 50 μg/mL propidium iodide in the dark at room temperature for 30 min, and then immediately analyzed by flow cytometry (FACSCalibur flow cytometer; BD Biosciences, San Jose, CA, USA).

### 2.8. ROS Detection

ROS was detected using an ROS assay kit (cat. no. S0033S, Beyotime Biotechnology, Shanghai, China) according to the manufacturer’s instructions and analyzed by flow cytometry (FACSCalibur flow cytometer, BD, San Jose, CA, USA), as previously described [24].

### 2.9. Xenograft Experiments

Six-week-old male BALB/c nude mice were purchased from Model Animal Research Center of Nanjing University and housed under pathogen-free conditions (12/12 h light/dark cycle, 24 ± 2 °C; humidity, 50 ± 10%) with free access to food and water. All animals were acclimated for at least 5–7 days before treatment. The xenograft of CRC was constructed using SW480/NC and SW480/NNMT cell lines as previously described [24]. After subcutaneous injection with SW480/NC and SW480/NNMT cells for 10 days, mice were treated with 5-FU (30 mg/kg body weight dissolved in saline, intraperitoneal administration, every 2 days) and/or Cur (100 mg/kg body weight dissolved in 10% PEG, intragastric administration, every other day). Development of the tumors and weight loss were regularly monitored for 3 weeks. All of the mice were intraperitoneally injected with barbiturate (100 mg/kg body weight) and promptly sacrificed by cervical dislocation at the end of the experiment, and all tumors were harvested. Tumor volume was calculated according to V = (length × width^2^)/2.

### 2.10. TUNEL Assay

Tumor tissues were fixed, embedded, and sliced according to a standard protocol. Apoptotic cells in tumor tissue sections was analyzed by TUNEL assay using an in situ Apoptosis Detection kit (cat. No. 11684795910, Roche Diagnostics, Basel, Switzerland) following the manufacturer’s instructions, as previously described [7]. Ten random fields of each tumor slide were selected, and the rate of positive stained cells was calculated.

### 2.11. Statistical Analysis

GraphPad Prism v7.0 software was used for statistical analysis. All data are presented as the mean ± SD from three independent experiments. The two-sample *t*-test was used for two-group comparisons. The ANOVA and Bonferroni method were used for multiple comparisons among more than two groups. *p* value < 0.05 was considered statistically significant difference.

## 3. Results

### 3.1. Cur Inhibits Cell Proliferation and Attenuates NNMT-Induced Resistance to 5-FU in CRC Cells

**Figure 1:**
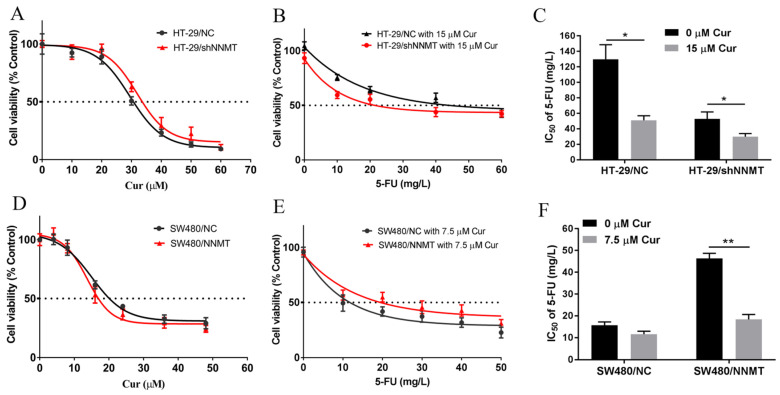
Cur inhibits CRC cell growth and attenuates NNMT-induced resistance to 5-FU. (**A**,**D**) CCK-8 analysis of HT-29/NC, HT-29/shNNMT, SW480/NC, and SW480/NNMT cell viability following treatment with Cur for 24 h. (**B**,**E**) CCK-8 analysis of HT-29/NC, HT-29/shNNMT, SW480/NC, and SW480/NNMT cell viability following treatment with Cur combined with different concentrations of 5-FU for 24 h. (**C**,**F**) IC_50_ of 5-FU when co-treated with or without Cur in HT-29/NC, HT-29/shNNMT, SW480/NC, and SW480/NNMT displayed as a histogram. 5-FU: 5-Fluorouracil, Cur: curcumin. Data are expressed as means ± SD, *n* = 3, * *p* < 0.05, ** *p* < 0.01.

The cytotoxic effects of Cur in human CRC cells SW480 and HT-29 were detected. SW480 exhibited low NNMT expression, while HT-29 showed a high level of NNMT. NNMT was knocked down in HT-29/shNNMT cells by NNMT-targeted shRNA with HT-29/NC as control. SW480/NNMT cells exogenously over-expressed NNMT with SW480/NC as control. HT-29/shNNMT, HT-29/NC, SW480/NNMT, and SW480/NC cells were treated with Cur for 24 h. A CCK-8 assay showed that the viability of CRC cells was inhibited by Cur, especially in high-NNMT-expression cell lines. The IC_50_ of Cur in the four cell lines is IC50 = 32.90 μM for HT-29/shNNMT, IC_50_ = 29.41 μM for HT-29/NC, IC_50_ = 13.69 μM for SW480/NNMT, and IC_50_ = 14.99 μM for SW480/NC (Figure 1A,D).

As previously reported, NNMT can reduce human CRC cells’ sensitivity to 5-FU [7]; thus, we aimed to determine whether Cur could reduce the NNMT-induced resistance to 5-FU in CRC cells. When we used the IC_50_ concentration of Cur combined with different concentrations of 5-FU, a considerable number of cells died, and it was difficult to evaluate the IC_50_ of 5-FU. Then, we used a lower concentration of Cur (15 μM in HT-29 cell lines and 7.5 μM in SW480 cell lines) to co-treat with 5-FU, and the IC_50_ of 5-FU was calculated using the CCK-8 assay. Table 1 showed that the IC_50_ of 5-FU was dramatically reduced when combined with Cur: from 131.81 to 50.98 mg/L in HT-29/NC (fold decrease of approximately 2.59), from 54.79 to 29.95 mg/L in HT-29/shNNMT (fold decrease of approximately 1.83) (Figure 1B,C), from 15.52 to 11.64 mg/L in SW480/NC (fold decrease of approximately 1.33), and from 46.32 to 18.2 mg/L in SW480/NNMT (fold decrease of approximately 2.55) (Figure 1E,F). In summary, it is suggested that Cur could inhibit cell proliferation and attenuate NNMT-induced resistance to 5-FU in CRC cells.

### 3.2. Cur Inhibits NNMT and p-STAT3 in CRC Cells

**Figure 2:**
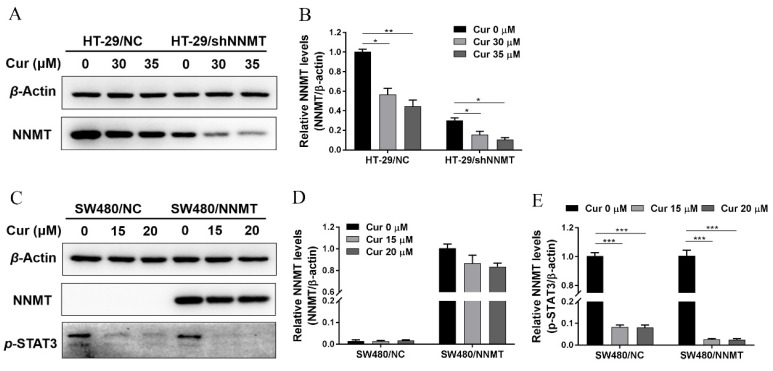
Cur downregulates NNMT and p-STAT3 in CRC cells. (**A**,**B**) NNMT proteins were determined by Western blot and displayed as a histogram after treatment with Cur for 24 h in HT-29 cells. The untreated group of HT-29/NC was used as the control group, which was set as 1 in the histogram. (**C**) NNMT and p-STAT3 proteins were determined by Western blot after treatment with Cur for 24 h in SW480 cells. (**D**) NNMT proteins were displayed as a histogram after treatment with Cur for 24 h in SW480 cells. The untreated group of SW480/NNMT was used as the control group, which was set as 1 in the histogram. (**E**) p-STAT3 proteins were displayed as a histogram after treatment with Cur for 24 h in SW480 cells. The untreated group of SW480/NC was used as the control group, which was set as 1 in the histogram. Cur: curcumin. Data are expressed as means ± SD, *n* = 3, * *p* < 0.05, ** *p* < 0.01, *** *p* < 0.001.

p-STAT3 was reported as an upstream transcription factor of NNMT, and Cur could depress p-STAT3 and then depress NNMT expression at the mRNA level [22]. Herein, we treated cells with two concentrations of Cur, 30 μM or 35 μM for HT-29 cell lines, which was approximately IC_50_ of HT-29/NC or HT-29/shNNMT, and 15 μM or 20 μM for SW480 cell lines, which was approximately IC_50_ of SW480/NNMT or SW480/NC. Following the treatment of cells with Cur, Western blot showed that NNMT was downregulated in HT-29 cell lines (Figure 2A,B), while in SW480 cell lines, no significant decrease in NNMT was detected (Figure 2C,D).

We also attempted to verify whether Cur inhibited NNMT expression by depressing p-STAT3. As our study previously reported, p-STAT3 is barely detected in HT-29 cell lines [24]. In SW480 cell lines, Cur could dramatically decrease p-STAT3 (Figure 2C,E). Collectedly, it is suggested that Cur could downregulate the endogenous expression of NNMT (in HT-29 cells) and p-STAT3 (in SW480 cells), but we cannot draw a conclusion that the inhibition of NNMT is due to the inhibition of p-STAT3 in CRC cells.

### 3.3. Cur Induces G2/M Phase Cell Cycle Arrest Especially in High-NNMT-Expression CRC Cells

**Figure 3:**
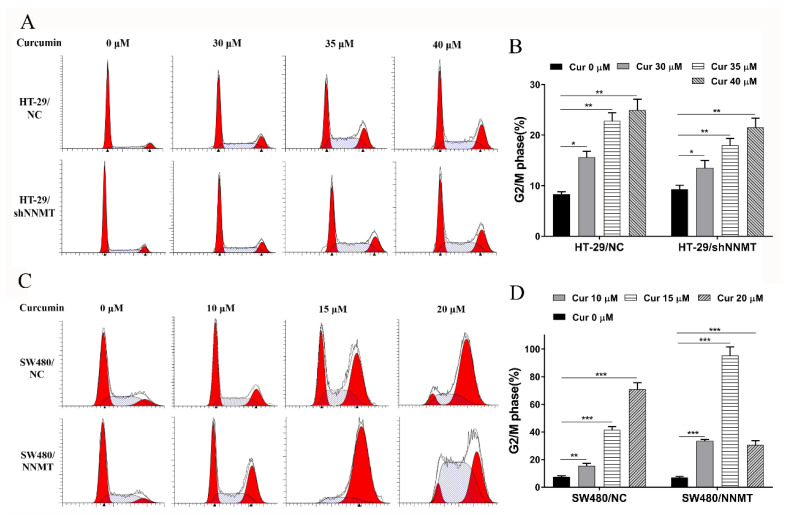
Cur induces cell cycle arrest in a dose-dependent manner in CRC cells. Cells were treated with Cur at different concentrations for 24 h. (**A**,**B**) Cell cycles were analyzed by flow cytometry and were displayed as a histogram in HT-29 cell lines. (**C**,**D**) Cell cycles were analyzed by flow cytometry and were displayed as a histogram in SW480 cell lines. Cur: curcumin. Data are expressed as means ± SD, *n* = 3, * *p* < 0.05, ** *p* < 0.01, *** *p* < 0.001.

It was investigated whether the anti-proliferative effect of Cur was associated with cell cycle arrest. Following treatment with different concentrations of Cur for 24 h, flow cytometry analysis showed that the proportion of cells at the G2/M phase was increased with increase in Cur concentration, especially in high-NNMT-expression cell lines. In HT-29 cell lines, the proportion of cells at the G2/M phase increased from 8.3 to 15.63% (30 μM Cur), to 22.79% (35 μM Cur), and to 24.95% (40 μM Cur) in HT-29/NC, and from 9.31 to 13.5% (30 μM Cur), to 17.97% (35 μM Cur), and to 21.53% (40 μM Cur) in HT-29/shNNMT (Figure 3A,B). Dramatic G2/M phase arrest was also found in SW480 cell lines, with the proportion of cells at the G2/M phase increased from 7.42 to 15.38% (10 μM Cur), to 41.43% (15 μM Cur), and to 71.01% (20 μM Cur) in SW480/NC, and from 6.93 to 33.44% (10 μM Cur), to 95.43% (15 μM Cur), and to 30.7% (20 μM Cur) in SW480/NNMT (Figure 3C,D).

**Figure 4:**
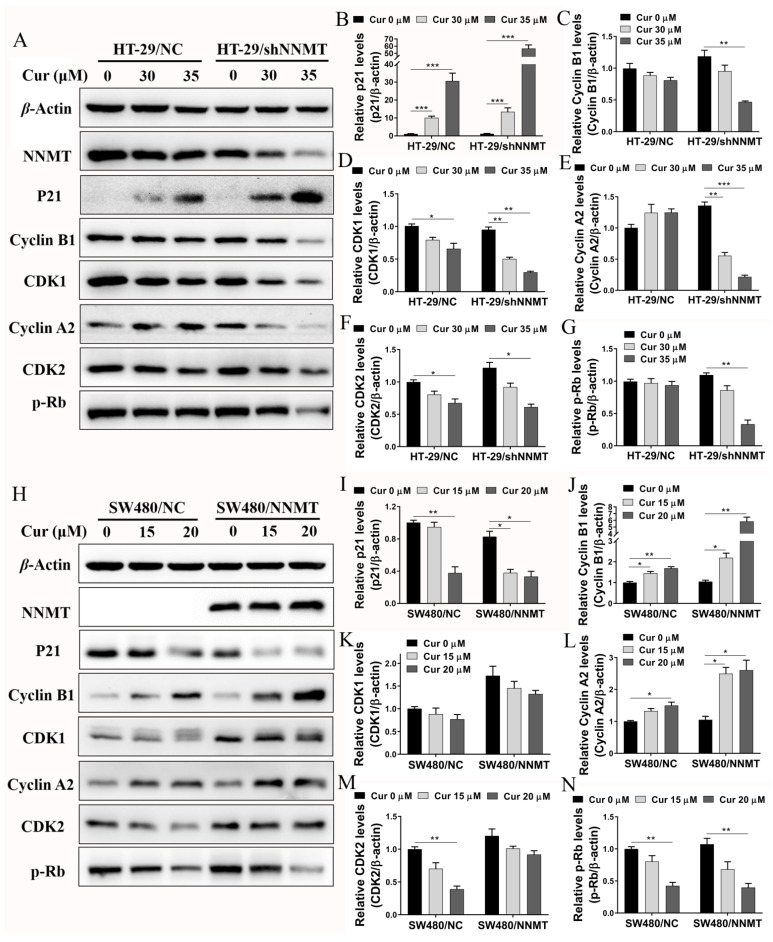
Cur regulates proteins related to the G2/M phase in CRC cells. Cells were treated with Cur at different concentrations for 24 h. (**A**–**G**) Proteins were analyzed by Western blot and were displayed as a histogram in HT-29 cell lines. The untreated group of HT-29/NC was used as the control group, which was set as 1. (**H**–**N**) Proteins were analyzed by Western blot and were displayed as a histogram in SW480 cell lines. The untreated group of SW480/NC was used as the control group, which was set as 1. Cur: curcumin. Data are expressed as means ± SD, *n* = 3, * *p* < 0.05, ** *p* < 0.01, *** *p* < 0.001.

The expression of G2/M phase-related proteins was detected after cells were treated with Cur. Western blot shows that the cell cycle inhibitory protein p21 was upregulated, while the cyclin-dependent kinases CDK1 and CDK2 were downregulated in a Cur-dependent manner in HT-29/NC and HT-29/shNNMT cell lines (Figure 4A,B,D,F). Although slight differences were detected in cyclin A2, cyclin B1, and p-Rb in HT-29/NC, significant decreases were found when NNMT was downregulated in HT-29/shNNMT (Figure 4A,C,E,G). Consistently, in SW480/NC and SW480/NNMT, p-Rb was significantly downregulated, and CDK1 and CDK2 were slightly decreased (Figure 4H,K,M,N). However, inconsistently, p21 was reduced, and cyclin A2 and cyclin B1 were increased in SW480 cell lines (Figure 4H–J,L). Thus, it was suggested that Cur could affect the cell cycle-related proteins and, in turn, induce the G2/M phase arrest in CRC cells.

### 3.4. Cur Has a Synergistic Effect with 5-FU via Induction of Cell Cycle Arrest in CRC Cells

**Figure 5:**
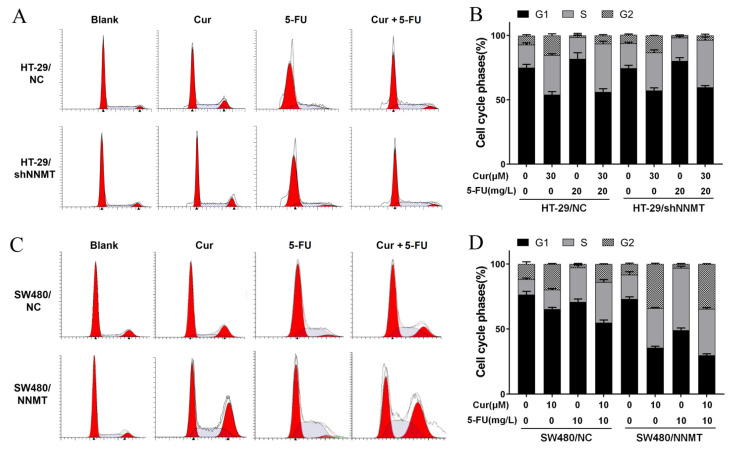
Cur combined with 5-FU induces cell cycle arrest in CRC cells. Cells were treated with Cur and/or 5-FU for 24 h. The untreated groups are displayed as blank groups. (**A**,**B**) Cell cycles were analyzed by flow cytometry and are displayed as a histogram in HT-29 cell lines. (**C**,**D**) Cell cycles were analyzed by flow cytometry and are displayed as a histogram in SW480 cell lines. 5-FU: 5-Fluorouracil, Cur: curcumin. Data are expressed as means ± SD, *n* = 3.

As previously mentioned, the combination of Cur with 5-FU yielded notable synergy in inhibiting cell proliferation, and it was also determined whether the synergistic effect with 5-FU is associated with cell cycle arrest. As when co-treated with a higher concentration of Cur and 5-FU, a considerable number of cells died, and it was difficult to conduct the following detection, we used 20 mg/L 5-FU in HT-29 cell lines and 10 mg/L 5-FU in SW480 cell lines, representing the half concentration of IC_50_ of 5-FU, combined with Cur (30 μM in HT-29 cells and 10μM in SW480 cells). Flow cytometry results show that 5-FU can dramatically induce G1/S phase arrest. The proportion of cells at the G1/S phase increased from 92.81 to 98.58% in HT-29/NC, from 93.83 to 98.23% in HT-29/shNNMT (Figure 5A,B, respectively), from 88.24 to 97.31% in SW480/NC, and from 91.71 to 96.92% in SW480/NNMT (Figure 5C,D, respectively). When co-treated with Cur and 5-FU, compared to cells treated with Cur alone, the proportion of cells at the G2/M phase was reduced in HT-29 cell lines, which could have been due to the G1/S phase arrest by 5-FU (Figure 5A,B). The unstuck cell cycle was also observed in SW480 cell lines following co-treatment with Cur and 5-FU, and it seems that the cells are arrested in both G1/S and G2/M (Figure 5C,D). We assume that Cur can reinforce the 5-FU-induced disorders on cell cycle in CRC cells.

**Figure 6:**
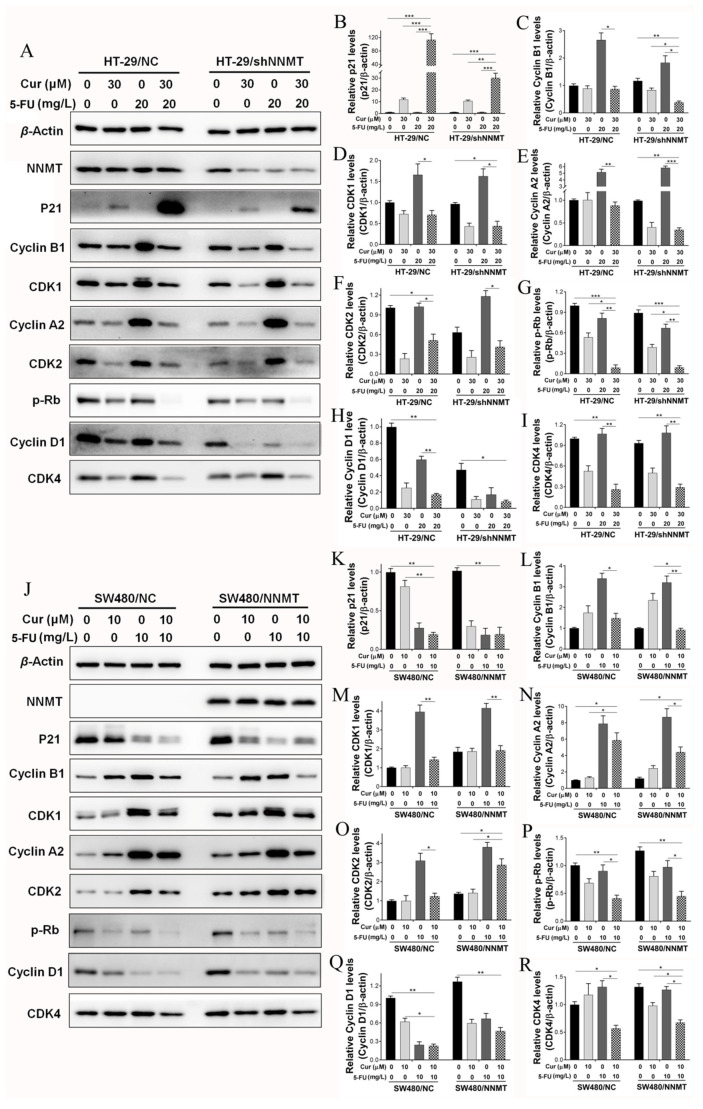
Cur and 5-FU regulate proteins related to cell cycle in CRC cells. Cells were treated with Cur (30 μM for HT-29 cells; 10 μM for SW480 cells) and/or 5-FU (20 mg/L for HT-29 cells; 10 mg/L for SW480 cells) for 24 h. (**A**–**I**) Proteins were analyzed by Western blot and are displayed as a histogram in HT-29 cell lines. The untreated groups of HT-29/NC were used as the control group, which was set as 1; (**J**–**R**) Proteins were analyzed by Western blot and are displayed as a histogram in SW480 cell lines. The untreated groups of SW480/NC were used as the control group, which was set as 1. 5-FU: 5-Fluorouracil, Cur: curcumin. Data are expressed as means ± SD, *n* = 3, * *p* < 0.05, ** *p* < 0.01, *** *p* < 0.001.

We further detected the cell cycle-related proteins after treatment with Cur combined with 5-FU. Western blot analysis showed that in HT-29 cells, p21 was more dramatically increased when co-treated with Cur and 5-FU than when treated with Cur or 5-FU alone (Figure 6A,B), and cyclin A2, cyclin B1, CDK1, and CDK2 were downregulated when co-treated with Cur and 5-FU, although 5-FU could increase them (Figure 6A,C–F). Both Cur and 5-FU can decrease p-Rb and the G1/S phase-related proteins CDK4 and cyclin D1, moreover, combination of 5-FU and Cur can reinforce the inhibition of p-Rb, CDK4 and cyclin D1 in HT-29 cells (Figure 6A,G–I). The cell cycle-related proteins were also detected in SW480 cell lines after treated with 10 μM Cur and/or 10 mg/L 5-FU for 24 h. Consistently, p-Rb and cyclin D1 could be decreased by Cur and 5-FU, and Cur had a synergistic effect with 5-FU on the inhibition of p-Rb and cyclin D1 in SW480 cells (Figure 6J,P,Q). Interestingly, although 5-FU could upregulate cyclin A2, cyclin B1, CDK1, CDK2, and CDK4 while Cur had a minimal effect on them, when co-treated with Cur and 5-FU, these proteins could be dramatically decreased in SW480 cells (Figure 6J,L–O,R). p21 decreased when treated with both Cur or 5-FU alone and when co-treated (Figure 6J,K). Thus we suggest that there is a considerable amount of synergy between Cur and 5-FU in terms of inducing cell cycle arrest in CRC cell lines.

### 3.5. Cur Induces Cell Cycle Arrest by Promoting ROS

**Figure 7:**
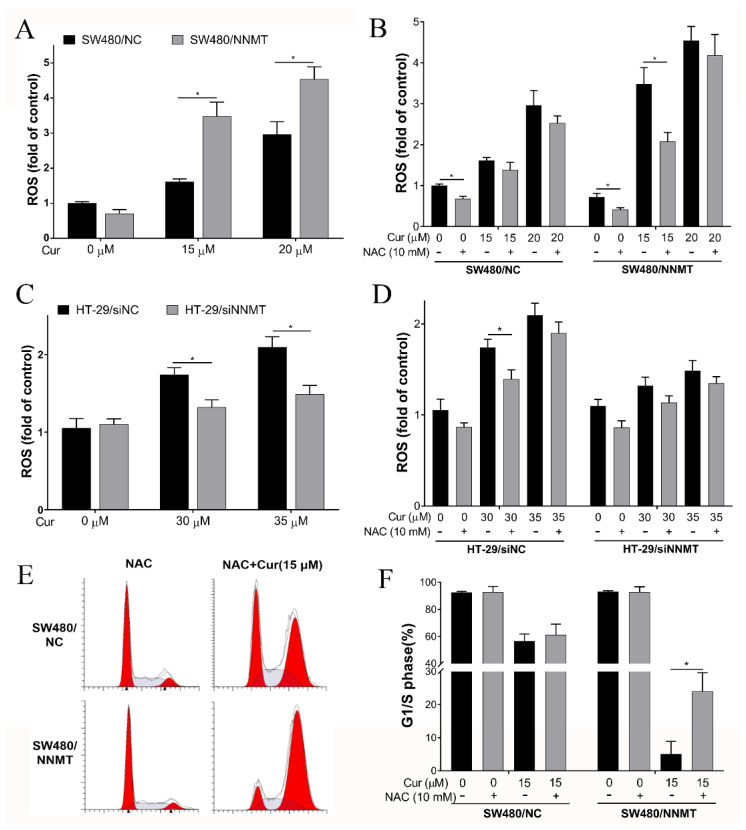
Cur induces cell cycle arrest by increasing ROS generation. Cells were pretreated with 10 mM NAC for 2 h and/or following treatment with Cur for 24 h. (**A**,**B**) ROS was detected by flow cytometry using the fluorescent probe DCFH−DA and is displayed as a histogram in SW480 cell lines. (**C**,**D**) ROS was detected by flow cytometry using the fluorescent probe DCFH−DA and is displayed as a histogram in HT-29 cell lines. (**E**,**F**) Cell cycles were detected by flow cytometry and are displayed as a histogram. Cur: curcumin. +: treated, −: untreated. Data are expressed as means ± SD, *n* = 3, * *p* < 0.05.

ROS is one of the causes of cell cycle arrest. It was determined whether Cur inducing cell cycle arrest through ROS in CRC cells. Herein, we found that Cur could promote ROS generation in SW480 cell lines, and the ROS level induced by Cur was higher in SW480/NNMT cells than in SW480/NC (Figure 7A). We used NAC, a known scavenger of ROS, to reverse the ROS, and flow cytometry showed that NAC pretreatment could reduce the ROS generation after incubation with 15 μM Cur, but when treated with 20 μM Cur, the ROS level induced by Cur was too high to be reduced (Figure 7B). Then, we used 15 μM Cur to detect the effect of NAC on cell cycles in SW480 cells. Following pretreatment with NAC, flow cytometry results showed that the G1/S phase was partially rescued in 15 μM Cur-treated SW480 cell lines, from 58.57 to 61.78% in SW480/NC and from 4.57 to 24.02% in SW480/NNMT (Figure 7E,F, respectively). The significant increase in G1/S by NAC is consistent with the decrease in ROS in SW480/NNMT (Figure 7B,F). These results show that ROS plays important roles in triggering the G2/M phase cell cycle arrest caused by Cur in SW480 cells. We have reported that NNMT could reduce ROS generation in CRC cells after treatment with 5-FU [7]. It is suggested that Cur could reverse the reduction of ROS by NNMT.

When we detected the ROS level induced by Cur in HT-29 cells, a pair of NNMT siRNA was used to knock down NNMT, as the lentiviral vector of NNMT shRNA used to construct the HT-29/NC and HT-29/shNNMT cell lines contained a green fluorescence protein tag that disturbs the green fluorescent signal of ROS. Consistent with the results in SW480 cell lines, the flow cytometry results show that following treatment with 30 μM or 35 μM Cur for 24 h, more ROS was induced in HT-29/siNC cells than in HT-29/siNNMT cells (Figure 7C). However, ROS promotion caused by Cur was much more mild than that in SW480 cell lines. Meanwhile, pretreatment with NAC could only slightly reduce the ROS promoted by Cur in HT-29 cells (Figure 7D), and the cell cycle arrest caused by Cur could not be reduced by NAC (data not shown).

### 3.6. Cur Has a Limited Synergetic Effect with 5-FU on Inducing Cell Apoptosis

**Figure A1:**
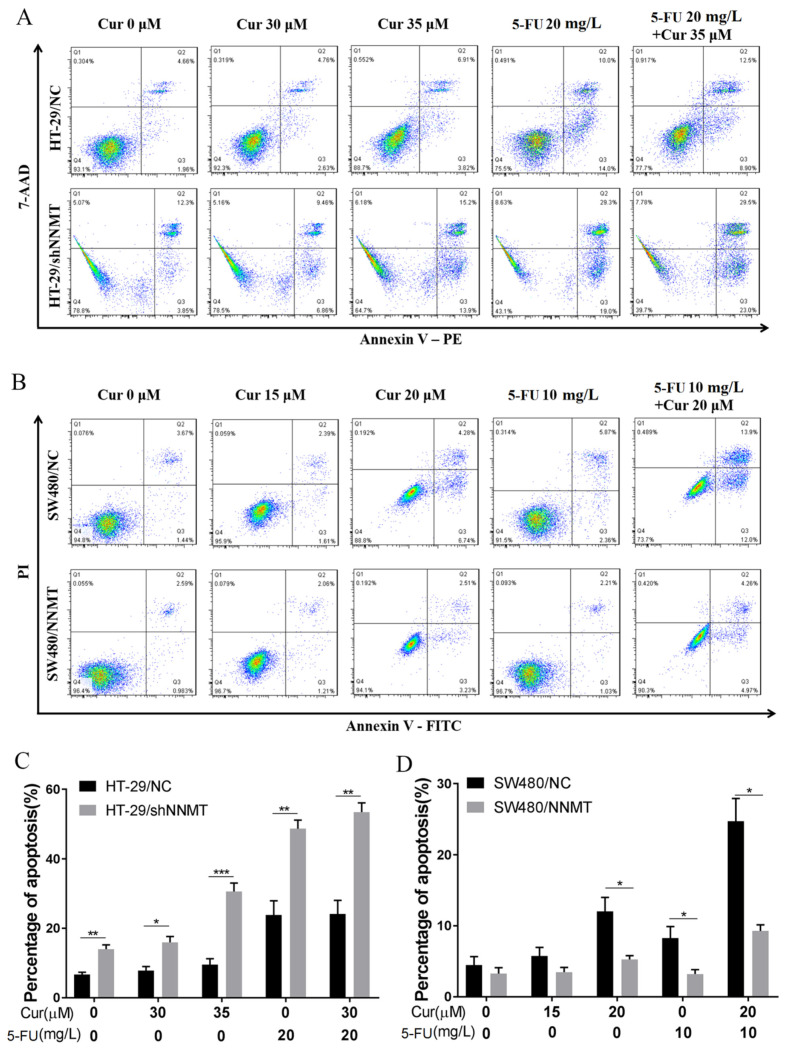
Cur combined with 5-FU induces cell apoptosis in CRC cells. Cells were treated with Cur (30 μM and 35 μM for HT-29 cells; 15 μM and 20 μM for SW480 cells) and/or 5-FU (20 mg/L for HT-29 cells; 10 mg/L for SW480 cells) for 24 h. (**A**,**C**) Cell apoptosis was analyzed by flow cytometry and displayed as a histogram of HT-29 cells. (**B**,**D**) Cell apoptosis was analyzed by flow cytometry and displayed as a histogram of SW480 cells. 5-FU: 5-Fluorouracil, Cur: curcumin. Data are expressed as means ± SD, *n* = 3, * *p* < 0.05, ** *p* < 0.01, *** *p* < 0.001.

Given that NNMT could induce resistance to 5-FU through reducing cell apoptosis in CRC cells [7], cell apoptosis was detected to investigate the effect of Cur on CRC cells with different NNMT expression levels. Following treatment with Cur (30 μM and 35 μM for HT-29 cells; 15 μM and 20 μM for SW480 cells) and/or 5-FU (20 mg/L for HT-29 cells; 10 mg/L for SW480 cells) for 24 h, flow cytometry showed that the proportion of cell apoptosis was increased. In HT-29 cell lines, the apoptosis increased from 6.68 to 7.81% (30 μM Cur), to 9.54% (35 μM Cur), to 23.87% (20 mg/L 5-FU), and to 24.1% (35 μM Cur and 20 mg/L 5-FU) in HT-29/NC, and from 13.99 to 15.97% (30 μM Cur), to 30.61% (35 μM Cur), to 48.66% (20 mg/L 5-FU), and to 53.46% (35 μM Cur and 20 mg/L 5-FU) in HT-29/shNNMT (Figure A1A,C). In SW480 cell lines, the apoptosis increased from 4.49 to 5.75% (15 μM Cur), to 12.04% (20 μM Cur), to 8.27% (10 mg/L 5-FU), and to 24.71% (20 μM Cur and 10 mg/L 5-FU) in SW480/NC, and from 3.27 to 3.47% (15 μM Cur), to 5.26% (20 μM Cur), to 3.21% (10 mg/L 5-FU), and to 9.27% (20 μM Cur and 10 mg/L 5-FU) in SW480/NNMT (Figure A1B,D).

The results indicate that apoptosis induced by Cur is much less in high-NNMT-expression cell lines than in low-NNMT-expression cell lines, and Cur has a limited synergistic effect with 5-FU on increasing cell apoptosis. We assume that NNMT could still protect CRC cells from apoptosis under Cur and/or 5-FU treatment.

### 3.7. Cur Has a Synergetic Effect with 5-FU on Decreasing Tumor Growth and Inducing Apoptosis In Vivo

**Figure 8:**
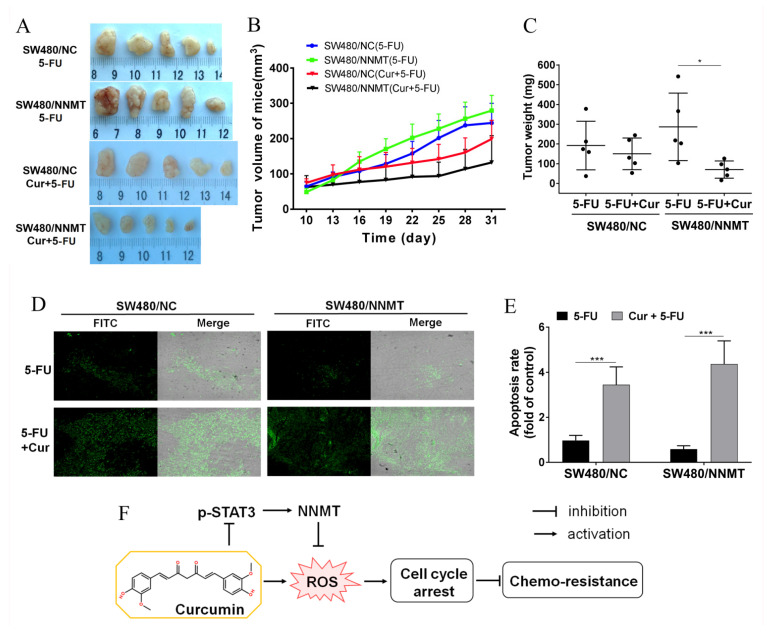
Cur inhibits tumor growth in a mouse xenograft model. (**A**) Representative picture showing tumors from subcutaneous tumor-bearing nude mice of SW480/NC or SW480/NNMT, groups which were treated with 5-FU and/or Cur. (**B**) Tumor proliferation curves calculated by tumor volume from day 10 to day 31. (**C**) Tumor weight of different groups displayed as a scatter diagram. (**D**) Cell apoptosis of tumor sections detected by TUNEL staining (×100). (**E**) Cell apoptosis rate calculated from TUNEL staining displayed as a histogram. The SW480/NC group treated with 5-FU was set as the control group and was set as 1. (**F**) Schematic illustration of Cur in the regulation of NNMT and chemo-sensitivity in CRC cells. Cur downregulates NNMT and p-STAT3 and enhances sensitivity to 5-FU via ROS-induced cell cycle arrest in CRC cells. 5-FU: 5-Fluorouracil, Cur: curcumin. Data are expressed as means ± SD, *n* = 3, * *p* < 0.05, *** *p* < 0.001.

Nude mice bearing SW480/NC and SW480/NNMT xenografts were used to explore the synergistic effect of Cur with 5-FU in vivo. We used 30 mg/kg 5-FU every 2 days for the 5-FU chemotherapy groups, as described in our previous study [7]. A previous study reported that up to 240 mg/kg/day is considered safe for mice [25]. In the current study, we used 100 mg/kg/day Cur for the 5-FU combination groups. It was observed that the average tumor volume and weight were smaller in the 5-FU combined with Cur treatment groups than in the 5-FU-treated groups, both in SW480/NC and SW480/NNMT tumors (Figure 8A–C). Moreover, cell apoptosis of the tumor sections was analyzed by TUNEL assay, and the results showed that more apoptosis was induced in groups treated with Cur combined with 5-FU than in groups treated with 5-FU alone (Figure 8D,E).

In conclusion, Cur is found to enhance sensitivity to 5-FU via downregulating NNMT and ROS-induced cell cycle arrest in CRC. A schematic illustration of Cur downregulating NNMT and attenuating NNMT-induced resistance to 5-FU in CRC cells is shown in Figure 8F.

## 4. Discussion

Chemo-resistance is a key reason for the poor prognosis of colorectal cancer (CRC). NNMT is an enzyme of nicotinamide metabolism, and we have previously reported that NNMT could enhance resistance to 5-FU in CRC [7]. Therefore, identifying NNMT-targeting drugs and detecting their anticancer efficacies are important for the battle against 5-FU resistance in CRC.

Given that NNMT is an enzyme that promotes cell proliferation and 5-FU resistance, inhibition of NNMT may be one of the ways to reduce 5-FU resistance. We have recently screened a natural products library and found that vanillin can decrease NNMT expression and attenuate NNMT-related resistance to 5-FU in CRC cells [24]. Curcumin (Cur), a promising natural compound of the turmeric plant, has been widely used in food. STAT3 is reported to upregulate NNMT expression, and Cur can inhibit NNMT expression in RNA levels as an inhibitor of p-STAT3 [22]. In the current study, it was also found that Cur could downregulate p-STAT3 and the endogenous expression of NNMT in protein levels. However, as NNMT is endogenously highly expressed and p-STAT3 is barely detected in HT-29 cells, in addition to NNMT being barely detected and p-STAT3 endogenously highly expressed in SW480 cells, we cannot conclude that the inhibition of NNMT by Cur is due to the inhibition of p-STAT3 in CRC cells, and we have not yet found a CRC cell line that possess high expression of both NNMT and p-STAT3. The precise mechanisms by which Cur inhibits NNMT expression require further research.

Cur has been reported to exert multiple anticancer effects [11,12,13,14]. In the present study, we also found that Cur can inhibit cell proliferation in CRC cells especially in high-NNMT-expression CRC cell lines and attenuate NNMT-induced resistance to 5-FU. To find the reasons that Cur caused cell death in CRC cells, especially in high-NNMT-expression cell lines, we detected the change of cell apoptosis and cell cycles following treated with Cur and/or 5-FU. In agreement with previous studies that Cur can induce apoptosis in CRC [17], it is found that Cur can induce cell apoptosis both in HT-29 and SW480 cell lines, and Cur has synergistic effect with 5-FU in increasing cell apoptosis. However, the apoptosis induced by Cur is much less in high-NNMT-expression cell lines than in low-NNMT-expression cell lines. Given that NNMT could induce resistance to 5-FU through reducing cell apoptosis in CRC cells [7], we assume that NNMT could also protect CRC cells from apoptosis by Cur.

In terms of the detection of cell cycles, it is found that Cur can induce more G2/M phase arrest in high-NNMT-expression cell lines than that in low ones, which is consistent with the CCK-8 assay results. Therefore, we deem that Cur reverse the NNMT-induced cell proliferation mainly through inducing cell cycle arrest rather than inducing cell apoptosis. Another important consideration is that subG1 changes is a marker of apoptosis and long exposures (at least 48 h) to some apoptosis-inducible drugs may lead to subG1 accumulation [26,27,28]. However, although Cur could induce apoptosis, no subG1 accumulation was detected following the Cur treatment for 24 h in the present study. We speculated that it might be related to the duration of Cur exposure. This phenomenon of no subG1 but apoptosis status had also been reported in several studies [29,30]. To identify the molecular mechanism by which Cur induces the G2/M phase arrest, we detected the proteins associated with the G2/M phase. Cells express different cyclins during the different stages of the cell cycle. CDK2 is activated by cyclin A2 during the late stages of DNA replication to drive the transition from S phase to G2 phase. Moreover, at the G2 phase, cyclin B complexes with CDK1 to facilitate the onset of mitosis [31]. CDKs phosphorylate and inactivate the retinoblastoma protein (Rb), an adaptor protein that represses transcription, which in turn controls the activity of E2F transcription factors that stimulate proliferation [32]. The cyclin-dependent kinase inhibitor p21 inhibits cell cycle progression primarily through the inhibition of CDK2 activity, and p21 disrupts the interaction between CDK and substrates that bind to CDK–cyclin [33]. Cur markedly decreases CDK1 in human glioma cells and downregulates CDK1 and cyclin B1 in human colon cancer Colo 205 cells [34,35]. In the current study, we found that Cur upregulates p21 and downregulates CDK1, CDK2, cyclin A2, cyclin B1, and p-Rb in HT-29 cells. However, in SW480 cells, Cur causes the G2/M phase arrest via significant inhibition of p-Rb and a slight decrease in CDK1 and CDK2. We assume that Cur plays different roles in different CRC cell lines, as this study lacks observations of a further mechanism.

Given that NNMT has previously noted to promote the cell cycle via the active G1 phase in CRC cells [6], and high expression of NNMT in both HT-29/NC and SW480/NNMT cells can protect CDK1, CDK2, cyclin A2, cyclin B1, and p-Rb against inhibition by Cur, which indicates that NNMT plays a role in promoting G2/M phase. This is inconsistent with the phenotype in which G2/M phase arrest is induced by Cur in high-NNMT-expression cell lines. In terms of inhibiting the cell cycle, in addition to affecting the interphase, Cur has also been reported to inhibit mitosis, including suppression of the spindle [36] and inhibition of mitotic kinesin Eg5 [37]. Besides, Cur has been identified as a DNA topoisomerase II poison involved in DNA damage [38,39]. As no reports have revealed the effect of NNMT on the inhibition of mitosis and DNA topoisomerase, the Cur’s impact on them may provide some clues as to why it can inhibit cells with high a NNMT expression. We assumed that NNMT might accelerate the cell cycle and Cur might be more effective at inhibiting cells that have a faster cell cycle. Further research is needed to confirm this.

Moreover, it was found that Cur has a synergistic effect with 5-FU on inducing cell cycle arrest in CRC cells. 5-FU is a pyrimidine analogue and can cause cell cycle arrest at the G1/S phase by inhibiting thymidylate synthetase [40]. In the current study, it was also noted that 5-FU could induce G1/S phase arrest in HT-29 and SW480 cell lines. Although the flow cytometry results show the unstuck cell cycles when co-treated with Cur and 5-FU, the Western bolt results show the dramatic synergistic effect of Cur and 5-FU on the inhibition of p-Rb, cyclins, and CDKs, thus, we assume that Cur can reinforce the disorders induced by 5-FU on the cell cycle in CRC cells.

ROS is one of the causes of cell cycle arrest, and previous studies have reported that Cur could induce ROS generation in CRC cells [41,42]. In this study, we also found that Cur can induce ROS in HT-29 and SW480 cell lines. NNMT is reported to reduce ROS generation in CRC cells after treatment with 5-FU [7], which means that NNMT might act as a ROS scavenger or inhibitor. Interestingly, it was found that Cur can induce more ROS generation in high-NNMT-expression CRC cell lines than in low-NNMT-expression ones. This might explain the more mortality of cells and G2/M phase arrests induced by Cur in high-NNMT-expression cells. A similar phenotype was also found in vanillin-treated CRC cells, in which vanillin induced more ROS and cell death in high-NNMT-expression cell lines [24]. However, further research is required to shed light on this seemingly contradictory phenomenon and to explain why the inhibition on ROS of NNMT disappeared. In this study, we also attempted to reduce ROS via NAC to determine whether ROS is one of the key causes of cell cycle arrest induced by Cur. It was found that NAC pretreatment can partially reduce the cell cycle arrest induced by Cur in SW480 cell lines. In HT-29, NAC could reduce the Cur-induced ROS generation, but only a fraction of the cell cycle arrest induced by Cur could be reduced by NAC, which is consistent with previous reports [16]. Moreover, the source of ROS generation and its mechanisms are not clear, and thus, warrant further research.

We constructed a murine xenograft model using SW480/NC and SW480/NNMT cells to assess the synergistic effect of Cur on 5-FU in vivo. It was observed that Cur could enhance the inhibition of 5-FU in tumor proliferation in both SW480/NC and SW480/NNMT groups. Consistent with a previous study [7], tumors in the SW480/NNMT groups exhibited a larger volume and less 5-FU-induced cell apoptosis than those of tumors in the SW480/NC groups when treated with 5-FU alone. However, when treated with Cur combined with 5-FU, it was noted that tumors in the SW480/NNMT were smaller, and there was less 5-FU-induced cell apoptosis when compared with tumors in the SW480/NC groups, which indicates that Cur exerts a stronger synergistic effect with 5-FU on high-NNMT-expression tumors than low-NNMT-expression tumors. This is inconsistent with the results of the in vitro cell experiments and could be due to the limited number of animals, leading to a statistical bias.

## 5. Conclusions

In the present study, it is demonstrated that Cur can reverse NNMT-induced resistance to 5-FU in vivo and in vitro, and the mechanism that involved in ROS generation and cell cycle arrest induced by Cur is partially demonstrated. Considering the fact that Cur has long been used, in this study, we propose Cur as a promising anticancer compound with minimal side effects for CRC adjuvant chemotherapy, particularly for CRC with high NNMT expression.

## Figures and Tables

**Table 1:** IC_50_ of 5-FU in CRC cell lines. The concentrations of Cur are 15 μM for HT-29 cell lines and 7.5 μM for SW480 cell lines. 5-FU: 5-Fluorouracil, Cur: curcumin.

Cell Lines	IC_50_ of 5-FU (mg/L)	IC_50_ of 5-FU under Cur (mg/L)	*p* Value
HT-29/shNC	131.81 ± 11.91	50.98 ± 4.21	0.023
HT-29/shNNMT	54.79 ± 4.39	29.95 ± 2.83	0.048
SW480/NC	15.52 ± 1.09	11.64 ± 1.06	0.110
SW480/NNMT	46.32 ± 1.66	18.2 ± 1.58	0.007

## Data Availability

Data are contained within the article.

## Appendix A

Cell apoptosis is detected to investigate the effect of curcumin (Cur) on Colorectal Cancer (CRC) cells with different NNMT expression levels. Following treatment with Cur (30 μM and 35 μM for HT-29 cells; 15 μM and 20 μM for SW480 cells) and/or 5-Fluorouracil (5-FU) (20 mg/L for HT-29 cells; 10 mg/L for SW480 cells) for 24 h, flow cytometry showed that the proportion of cell apoptosis increases. In HT-29 cell lines, the apoptosis increases from 6.68 to 7.81% (30 μM Cur), to 9.54% (35 μM Cur), to 23.87% (20 mg/L 5-FU), and to 24.1% (35 μM Cur and 20 mg/L 5-FU) in HT-29/NC, and from 13.99 to 15.97% (30 μM Cur), to 30.61% (35 μM Cur), to 48.66% (20 mg/L 5-FU), and to 53.46% (35 μM Cur and 20 mg/L 5-FU) in HT-29/shNNMT (Figure A1A,C). In SW480 cell lines, the apoptosis increases from 4.49 to 5.75% (15 μM Cur), to 12.04% (20 μM Cur), to 8.27% (10 mg/L 5-FU), and to 24.71% (20 μM Cur and 10 mg/L 5-FU) in SW480/NC, and from 3.27 to 3.47% (15 μM Cur), to 5.26% (20 μM Cur), to 3.21% (10 mg/L 5-FU), and to 9.27% (20 μM Cur and 10 mg/L 5-FU) in SW480/NNMT (Figure A1B,D). The results indicate that Cur has a limited synergistic effect with 5-FU on increasing cell apoptosis. Given that the apoptosis induced by Cur is much less in high-NNMT-expression cell lines than in low-NNMT-expression cell lines, we assume that NNMT could still protect CRC cells from apoptosis under Cur and/or 5-FU treatment.
